# Genomic findings of hypertrophic and dilated cardiomyopathy characterized in a Thai clinical genetics service

**DOI:** 10.1371/journal.pone.0267770

**Published:** 2022-09-27

**Authors:** Objoon Trachoo, Teerapat Yingchoncharoen, Tawai Ngernsritrakul, Nareenart Iemwimangsa, Bhakbhoom Panthan, Sommon Klumsathian, Sasima Srisukh, Anucha Mukdadilok, Sithakom Phusanti, Angkana Charoenyingwattana, Takol Chareonsirisuthigul, Wasun Chantratita, Tarinee Tangcharoen

**Affiliations:** 1 Faculty of Medicine Ramathibodi Hospital, Department of Medicine, Mahidol University, Bangkok, Thailand; 2 Faculty of Medicine Ramathibodi Hospital, Center for Medical Genomics, Mahidol University, Bangkok, Thailand; 3 Faculty of Medicine Ramathibodi Hospital, Department of Pathology, Mahidol University, Bangkok, Thailand; University of Florida, UNITED STATES

## Abstract

Hypertrophic cardiomyopathy (HCM) and dilated cardiomyopathy (DCM) are the most common referrals in the Inherited Cardiovascular Condition (ICC) Genetics Service. Several issues must be discussed with patients and their families during the genetic consultation session, including the options for genetic testing and cardiovascular surveillance in family members. We developed an ICC registry and performed next-generation-based DNA sequencing for all patients affected by non-syndromic HCM and idiopathic DCM in our joint specialist genetics service. The target gene sequencing panel relied on the Human Phenotype Ontology with 237 genes for HCM (HP:0001639) and 142 genes for DCM (HP:0001644). All subjects were asked to contact their asymptomatic first-degree relatives for genetic counseling regarding their risks and to initiate cardiovascular surveillance and cascade genetic testing. The study was performed from January 1, 2014, to December 31, 2020, and a total of 62 subjects (31-HCM and 31-DCM) were enrolled. The molecular detection frequency was 48.39% (32.26% pathogenic/likely pathogenic, 16.13% variant of uncertain significance or VUS for HCM, and 25.81% (16.13% pathogenic/likely pathogenic, 9.68% VUS) for DCM. The most prevalent gene associated with HCM was *MYBPC3*. The others identified in this study included *ACTN2*, *MYL2*, *MYH7*, *TNNI3*, *TPM1*, and *VCL*. Among the DCM subjects, variants were detected in two cases with the *TTN* nonsense variants, while the others were missense and identified in *MYH7*, *DRSP3*, *MYBPC3*, and *SCN5A*. Following the echocardiogram surveillance and cascade genetic testing in the asymptomatic first-degree relatives, the detection rate of new cases was 8.82% and 6.25% in relatives of HCM and DCM subjects, respectively. Additionally, a new pre-symptomatic relative belonging to an HCM family was identified, although the genomic finding in the affected case was absent. Thus, ICC service is promising for the national healthcare system, aiming to prevent morbidity and mortality in asymptomatic family members.

## Introduction

An inherited cardiovascular condition (ICC) is one of the most common referrals in clinical genetics services. ICC requires the efforts of a multidisciplinary team to serve patients and their families and to prevent morbidity and mortality in at-risk family members. The most common ICCs in public health include cardiomyopathy, aortopathy, pulmonary hypertension, and arrhythmias [1–4]. In many developing countries, such as Thailand and other Southeast Asian nations, clinical genetic services are limited owing to a limited number of clinical geneticists, laboratory geneticists, and genetic counselors working in this field [5–8]. Few medical centers have established joint specialist clinics between cardiologists and geneticists to provide ICC genetic services for patients and families to receive genetic consultation, counseling, and targeted genetic testing where indicated. Our retrospective data from our medical school in Bangkok, a tertiary medical care setting, revealed that the most common ICC referrals are hypertrophic cardiomyopathy (HCM) and dilated cardiomyopathy (DCM), which usually result in serious medical outcomes.

HCM is defined as left ventricular hypertrophy (LVH) in the absence of abnormal loading. Several studies have consistently reported a prevalence of unexplained LVH in approximately 1 in 500 adults worldwide [9,10]. The most common forms are familial and inherited by autosomal dominant patterns caused by the mutations in genes encoding cardiac sarcomere protein [11–14]. Less than 10% are associated with the inborn error of metabolism, neuromuscular disorders, and malformation syndromes [15,16].

The clinical manifestations of HCM range from asymptomatic to progressive heart failure and sudden cardiac death. The symptoms vary from individual to individual, even within the same family. Common symptoms include shortness of breath on exertion, chest pain, palpitations, orthostasis, presyncope, and syncope [14]. LVH most often becomes apparent during adolescence or young adulthood [17]. However, LVH can develop later in life, even in infancy and childhood. As measured by Doppler echocardiographic imaging, diastolic dysfunction is a common finding in the overt disease [15]. Approximately 25% of persons with HCM have a detectable intracavitary obstruction at rest, but a much higher proportion may develop obstructive physiology with provocation [18–20]. Individuals with HCM are at an increased risk for atrial fibrillation (AF), a significant cause of morbidity in adults [21–23]. In addition, approximately 10%-20% have a lifetime-increased risk for sudden cardiac death (SCD) due to ventricular arrhythmia [24,25].

DCM is defined as a myocardial disorder characterized by the presence of LV dilatation and LV systolic impairment in the absence of abnormal loading conditions, such as hypertension, coronary artery disease, and valvular heart disease [26]. DCM prevalence is thought to be in the range of 1 in 2,500 adults, with an annual incidence of 5–8 per 100,000 [9]. In children, the incidence is much lower (0.5–0.8 per 100,000 per year) [27, 28].

DCM usually presents with one of the following symptoms: 1) congestive heart failure, 2) arrhythmias and/or conduction system defects, and 3) thromboembolic stroke and an asymptomatic condition found during annual check-ups [29]. The diagnosis is made by the presence of LV enlargement and systolic dysfunction assessed by two-dimensional echocardiography. An ejection fraction of less than 50% is considered systolic dysfunction. Fractional shortening is another clinical measure of systolic function. A fractional shortening of less than 25–30% is considered systolic dysfunction [30]. Other non-invasive studies can also facilitate the establishment of a diagnosis, such as cardiac nuclear studies, magnetic resonance imaging, and left ventricular angiography [31,32].

The etiology of DCM can be classified as genetics and acquired; up to 35% of DCM cases are genetics, including syndromic and non-syndromic forms. Syndromic DCM can be found in many conditions, including neuromuscular disorders, inborn errors of metabolism, and malformation syndromes [33]. After excluding all acquired identifiable causes, DCM is traditionally referred to as idiopathic dilated cardiomyopathy (IDC), which includes genetic forms of DCM. When two or more closely related family members meet a formal diagnostic standard for IDC (by excluding all detectable causes of DCM), the diagnosis of familial dilated cardiomyopathy (FDC) is made [34–36].

Currently, it is well known that genetic testing for cardiomyopathy is beneficial for disease management and helps family members in surveillance and early treatment. Herein, we present genetic data contributing to the two most common genetic referrals, HCM and DCM, and discuss the benefits of genetic testing.

## Materials and methods

### Ethics approval

The study was approved by the Committee on Human Rights Related to Research Involving Human Subjects, Faculty of Medicine Ramathibodi Hospital, Mahidol University, with documentary proof of ethical clearance no. MURA2011/506 entitled “Identification of genes causing hereditary cardiomyopathies in the Thai population” (approved April 11, 2013).

### Patient registry

All patients diagnosed with non-syndromic HCM and idiopathic DCM who received genetic consultation and counseling at the Joint Specialist Clinic between the Adult Cardiology Unit and Centre for Medical Genomics at the Faculty of Medicine Ramathibodi Hospital, Bangkok, Thailand, between January 1, 2014, and December 31, 2020, were enrolled in the ICC registry. The HCM and DCM diagnoses met the cardiac imaging criteria [37,38]. Informed consent was obtained for clinical data collection and genetic testing. For minors younger than twenty-years-old, informed consent was obtained by either their parents or guardians.

### Tracking of relatives

All asymptomatic first-degree relatives were informed to perform echocardiography and genetic surveillance by issuing referral letters to their primary healthcare providers within one year of the initial clinical diagnosis. There were two options for cardiovascular surveillance: 1) performing surveillance at the local cardiovascular service and bringing the referral letter describing the results back to the ICC clinic, or 2) referring relatives to cardiologists in the ICC clinic to perform surveillance. Individuals ≥ 12 years were given recommendations to undergo echocardiography and genetic testing, while children aged less than 12 years were only asked to be subjected to physical examination. Echocardiography and genetic testing were performed in younger children if abnormal cardiac findings were detected during a physical examination. Due to ethical considerations, genetic testing in the relatives was performed by Sanger’s DNA sequencing of the targeted variants, which were only characterized as pathogenic or likely pathogenic. Informed consent was obtained from all relatives before processing cardiovascular surveillance and targeted variant testing.

### Sample collection and DNA extraction

Peripheral venous blood samples were collected by using EDTA as an anticoagulant. DNA was extracted from leukocytes using an automatic nucleic acid isolation system (QuickGene-610L, Kurabo Industries, Japan).

### Next-generation-based DNA sequencing

**Fig 1:**
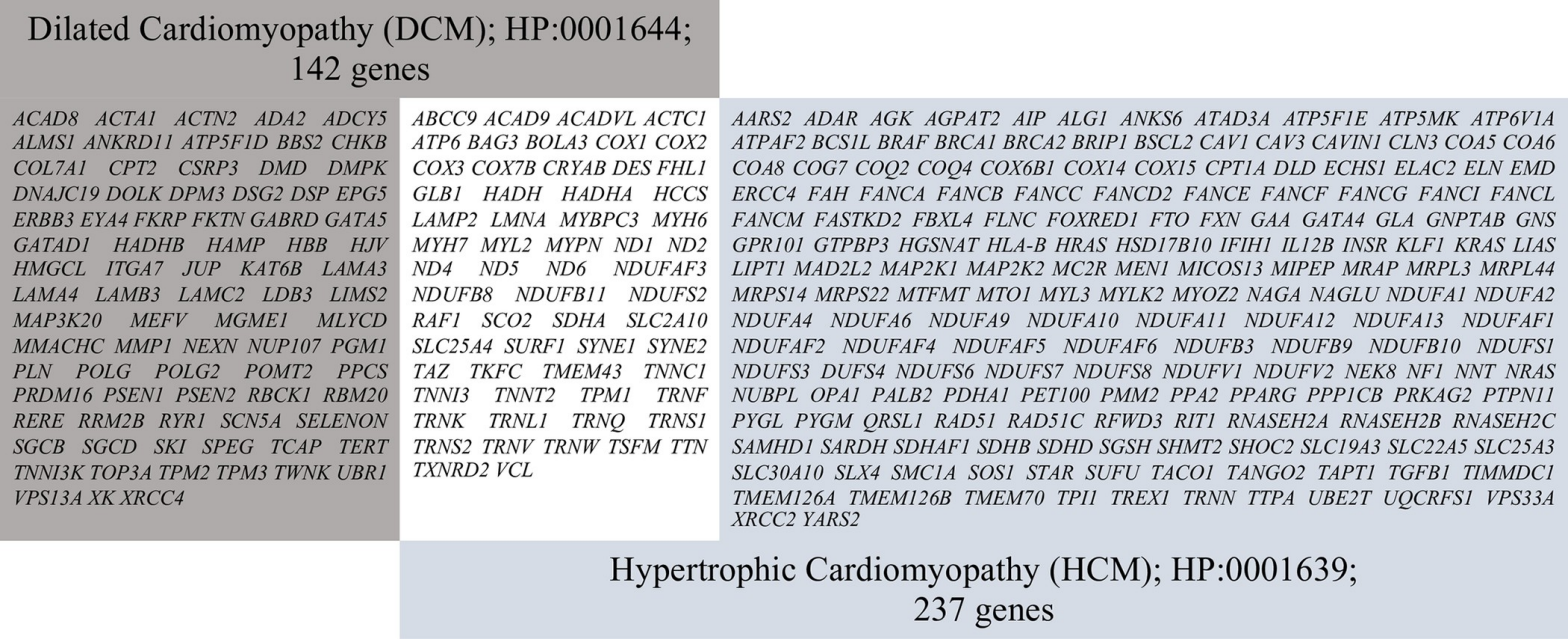
Targeted gene list for hypertrophic and dilated cardiomyopathy. Based on the Human Phenotype Ontology, 237 and 142 genes are listed as the genetic cause of hypertrophic cardiomyopathy (HP:0001639) and dilated cardiomyopathy (HP:0001644), respectively. Of them, 61 genes are described as the etiology of both phenotypes (white box).

Using genomic DNA from the submitted specimens, all exons and/or flanking splice junctions of genes in the target gene list were sequenced (Fig 1**).** Next-generation sequencing was performed using SureSelect Human All Exon V7 (Agilent Technology, Santa Clara, CA). The NGS library was prepared for paired-end (2 x 150 bp) sequencing on the NovaSeq 6000 platform (Illumina, San Diego, CA) according to the manufacturers’ recommendations. The DNA sequences were aligned to the human genome reference sequence (GRCh38/hg38 build) with GATK version 4.0 (Broad Institute, Cambridge, MA). The HaplotypeCaller was used for variant calling (Broad Institute). All variants obtained from the Sure Select Human All Exon kit 71Mb were obtained and subsequently analyzed within the region of 12 base distances from splice site boundaries. Sequencing results have an average coverage depth of about **100**x with more than **9**0**%** of the targeted bases achieved >2**0**x. For quality filtering, variants with reading depths >10× coverage in a single allele and >20× scope in homozygotes were selected. Each of the selected variants had a threshold quality score >Q40. Variant discovery analysis was performed using VarSeq version 2.2.1 (Golden Helix, Bozeman, MT). Candidate variants of specific genes were determined using a minor allele frequency (MAF) ≤ 0.05, East Asian (EAS) population data from the 1000 Genomes Project phase III, gnomAD Exomes Variant Frequencies 2.0.1, gnomAD Genomes Variant Frequencies 2.0.1, BROAD, and an in-house Thai Exome database (updated December 2020). Functional prediction based on dbNSFP Functional Predictions and Scores 3.0, GHI, and splice affecting was determined using prediction scores from dbscSNV Splice Altering Predictions 1.1, GHI [39].

Variant calling was based on the human phenotype ontology (HP:0001639 for HCM and HP:0001644 for DCM (Fig 1). The interpretation of sequence variants was based on HGMD professional 2020.3 release (The Human Gene Mutation Database, the Institute of Medical Genetics in Cardiff, UK), ClinVar database (updated on November 1, 2020; National Center for Biotechnology Information, USA National Library of Medicine), and the OMIM® database (updated on November 1, 2020; Online Mendelian Inheritance in Man®, Johns Hopkins University, USA). Variant classification relied on the standards and guidelines recommended by the American College of Medical Genetics and Genomics (ACMG) and the Association for Molecular Pathology (AMP) [40]. Sanger sequencing was confirmed in all variants in the panel, except for likely benign and benign characteristics. Variant interpretation for DCM further relied on ACMG/ClinGen guidelines for the DCM Precision Medicine study [41].

### Statistical analysis

Most data were presented using descriptive statistics. Comparisons between groups were performed using Fisher’s exact test when p < 0.05, which was accepted as statistically significant. Data analysis was performed using GraphPad® software (GraphPad, San Diego, CA).

## Results

### Basic clinical information of patients registered in inherited cardiovascular clinics

A total of 62 subjects (31-HCM and 31-DCM) were enrolled from January 1, 2014, to December 31, 2020 (8.86 cases/year). Among the patients in the HCM registry, the majority were male (*n* = 22; 70.97%). The average age of onset was 53.03 ± 15.87 years. The median age of onset was 52 years. Of these, 22 (70.97%) had a later onset at ≥ 45 years of age. Eighteen subjects (58.06%) had left ventricular obstruction as assessed by echocardiography, while the rest had non-obstructive or apical cardiomyopathy. Three patients (9.68%) reported that they had experienced an unexplained cause of syncope. Fifteen patients (48.39%) had experienced at least one of the following arrhythmias: atrial fibrillation, supraventricular tachycardia, ventricular tachycardia, or ventricular fibrillation. Implantable cardioverter-defibrillator (ICD) surgery was performed on seven patients (22.58%). A positive family history of hypertrophic cardiomyopathy or sudden unexplained cardiac death in one of the first- or second-degree relatives was observed in 16 patients (51.61%). Genomic variants related to HCM were detected in 15 cases (48.39%), of which 10 (32.26%) were classified as pathogenic/likely pathogenic (P/LP) variants, and the others (16.13%) were classified as VUS (S1 Table). Clinical factors related to the presence of genetic variants (P/LP and VUS) were age of onset less than the median age (*p* < 0.01; odds ratio 11.92; 95% CI 2.27–50.84) and presence of left ventricular obstruction (*p* < 0.05; odds ratio 6.67; 95% CI 1.32–27.42), while the other factors displayed no statistical significance (*p* > 0.05).

Regarding the DCM registry, the majority of the enrolled subjects were male (*n* = 21; 67.74%). The average onset was 42.77 ± 14.19 years. The median age of onset was 44 years. Fifteen patients (48.39%) had later onset at ≥ 45 years of age. The mean left ventricular ejection fraction was 31.32 ± 9.86%. Twenty patients (64.52%) experienced arrhythmia, as described above. Of them, 10 (32.26%) patients were treated with ICD implantation. A positive family history of dilated cardiomyopathy, sudden unexplained cardiac death, and unknown cause of congestive heart failure in first- or second-degree relatives was described in nine cases (29.03%). Genomic variants related to DCM were identified in eight cases (25.81%), of which five cases (16.13%) were classified as pathogenic/likely pathogenic, and the others (9.68%) were classified as VUS (S2 Table). None of the clinical factors were related to the presence of P/LP and VUS (*p* > 0.05).

### Genomic findings of hypertrophic and dilated cardiomyopathy

All genomic variants related to HCM and DCM and their essential bioinformatic parameters are summarized alphabetically (Table 1). *MYBPC3* variants were the most prevalent among HCM patients, while nonsense *TTN* variants accounted for the majority in the DCM registry.

**Table 1:** Summary of genomic findings of variants related to hypertrophic and dilated cardiomyopathy.

Genes	Phenotype (HCM/ DCM)	HGVS Coding DNA	HGVS Protein	Zyg	Variant Impact	dbSNP	Previous Report^b^ (yes/ no)	1000G MAF Global	1000G MAF East Asian	SIFT Prediction Score	Polyphen-2 Prediction Score	Mutation Tester Score	Conser	Final Classification
*ACTN2*	HCM	NM_001103.4:c.1586A>G	NP_001094.1:p.(Asn529Ser)	Het	Missense	rs200143657	Yes	N/A	N/A	Damaging	Probably damaging	Damaging	High	VUS
*CSRP3*	DCM	NM_003476.5:c.571G>A	NP_003467.1:p.(Glu191Lys)	Het	Missense	rs1417050043	Yes	N/A	N/A	Damaging	Probably damaging	Damaging	High	VUS
*MYBPC3*	HCM	NM_000256.3:c.1522C>T	NP_000247.2:p.(Gln508Ter)	Het	Nonsense	rs730880544	Yes	N/A	N/A	N/A	N/A	Damaging	High	Pathogenic
*MYBPC3*	HCM	NM_000256.3:c.3624_3624delC	NP_000247.2:p.(Lys1209fs)	Het	FS del	rs397516029	Yes	N/A	N/A	N/A	N/A	N/A	N/A	Pathogenic
*MYBPC3*	HCM	NM_000256.3:c.2864_2865delCT	NP_000247.2:p.(Pro955fs)	Het	FS del	rs397515990	Yes	N/A	N/A	N/A	N/A	N/A	N/A	Pathogenic
*MYBPC3*	HCM	NM_000256.3:c.1058delA	NP_000247.2:p.(Lys353Argfs*3)	Het	FS del	N/A	No	N/A	N/A	N/A	N/A	N/A	N/A	Pathogenic
*MYBPC3*	HCM	NM_000256.3:c.3190+5G>A^a^		Het	Splicing	rs587782958	Yes	N/A	N/A	N/A	N/A	N/A	N/A	Pathogenic
*MYBPC3*	HCM	NM_000256.3:c.2300A>G	NP_000247.2:p.(Lys767Arg)	Het	Missense	rs760786216	Yes	N/A	N/A	Tolerated	Possibly damaging	Damaging	High	Likely pathogenic
*MYBPC3*	HCM	NM_000256.3:c.1720C>T	NP_000247.2:p.(Arg574Trp)	Het	Missense	rs61897383	Yes	N/A	N/A	Damaging	Possibly damaging	Damaging	High	VUS
*MYBPC3*	HCM	NM_000256.3:c.1144C>G	NP_000247.2:p.(Arg382Gly)	Het	Missense	N/A	No	N/A	N/A	Damaging	Possibly damaging	Tolerated	High	VUS
*MYBPC3*	DCM	NM_000256.3:c.1246G>A	NP_000247.2:p.(Gly416Ser)	Het	Missense	rs371513491	Yes	N/A	N/A	Damaging	Probably damaging	Damaging	High	Likely pathogenic
*MYH7*	HCM	NM_000257.4:c.2146G>A	NP_000248.2:p.(Gly716Arg)	Het	Missense	rs121913638	Yes	N/A	N/A	Damaging	Probably damaging	Damaging	High	Pathogenic
*MYH7*	DCM	NM_000257.4:c.3157C>T	NP_000248.2:p.(Arg1053Trp)	Het	Missense	rs730880903	Yes	N/A	N/A	Damaging	Probably damaging	Damaging	High	Pathogenic
*MYH7*	DCM	NM_000257.4:c.4298A>G	NP_000248.2:p.(Glu1433Gly)	Het	Missense	N/A	No	N/A	N/A	Tolerated	Probably damaging	Damaging	High	VUS
*MYL2*	HCM	NM_000432.4:c.173G>A	NP_000423.2:p.(Arg58Gln)	Het	Missense	rs104894369	Yes	N/A	N/A	Tolerated	Probably damaging	Damaging	High	Pathogenic
*SCN5A*	DCM	NM_000335.5:c.677C>T	NP_000326.2:p.(Ala226Val)	Het	Missense	rs199473561	Yes	N/A	N/A	Damaging	Probably damaging	Damaging	High	VUS
*TNNI3*	HCM	NM_000363.5:c.370G>C	NP_000354.4:p.(Glu124Gln)	Het	Missense	rs727503506	Yes	N/A	N/A	Damaging	Probably damaging	Damaging	High	Pathogenic
*TNNT2*	DCM	NM_001276345.2:c.506G>A	NP_001263274.1:p.(Arg169Gln)	Het	Missense	rs45501500	Yes	N/A	N/A	Damaging	Probably damaging	Damaging	Low	Likely pathogenic
*TPM1*	HCM	NM_000366.6:c.343G>A	NP_000357.3:p.(Glu115Lys)	Het	Missense	rs727504313	Yes	N/A	N/A	Damaging	Probably damaging	Damaging	High	VUS
*TTN*	DCM	NM_001267550.2:c.85493G>A	NP_001254479.2: p.(Trp28498Ter)	Het	Nonsense	rs756499458	No	N/A	N/A	N/A	N/A	Damaging	High	Pathogenic
*TTN*	DCM	NM_001256850.1:c.71731C>T	NP_001243779.1: p.(Arg23911Ter)	Het	Nonsense	rs545954490	Yes	N/A	N/A	Damaging	N/A	Damaging	Low	Pathogenic
*VCL*	HCM	NM_003373.4:c.833A>G	NP_003364.1:p.(Asn278Ser)	Het	Missense	N/A	No	N/A	N/A	Tolerated	Benign	Damaging	High	VUS

^a^Amino acid changes cannot be defined in splicing variants.

^b^Previous reports were documented in ClinVar and HGMD databases (see materials and methods).

Abbreviations: HCM, hypertrophic cardiomyopathy; DCM, dilated cardiomyopathy; HGVS, Human Genome Variation Society; Zyg, zygosity; Het, heterozygosity; FS del, frameshift deletion; Conser, conservation score; N/A, not available.

### Surveillance in asymptomatic first-degree relatives

Of the 62 families, 36 (60%) followed the recommendation to undergo cardiovascular and genetic surveillance within six months after the first genetic consultation of the index cases. Among 66 first-degree relatives of 36 families, cardiomyopathy was identified in five (7.58%). HCM was newly diagnosed in three of 34 relatives (8.82%), and DCM was detected in two of 32 (6.25%) relatives (Table 2). Four relatives were reported of good health status, but their cardiac imaging findings met the diagnostic criteria for either HCM or DCM. Additionally, there was one family (H020) where the index case and the asymptomatic first-degree relative presented with significant echocardiogram findings, but the genomic variant was absent. On the other hand, our study did not detect any asymptomatic relatives who carried genomic variants without meeting cardiac imaging diagnostic criteria.

**Table 2:** Echocardiographic surveillance and the variants identified in asymptomatic first-degree relatives within six months following the first genetic consultation.

Registry	Total families	Number of families with at least one first-degree relative coming for genetic consultation and surveillance	Total number of relatives performing echocardiography	Newly diagnosed cases	Index case ID belonging to the newly diagnosed relatives	Genes	HGVS Coding DNA	HGVS Protein	Relatives who were newly diagnosed
HCM	31	18 (58.06%)	34	3 (8.82%)	H005	*MYBPC3*	NM_000256.3:c.3624_3624delC	NP_000247.2:p.(Lys1209fs)	A 29-year-old brother
					H014	*MYBPC3*	NM_000256.3:c.2300A>G	NP_000247.2:p.(Lys767Arg)	A 68-year-old mother
					H020	Negative			A 25-year-old son
DCM	31	18 (58.06%)	32	2 (6.25%)	D022	*MYBPC3*	NM_000256.3:c.1246G>A	NP_000247.2:p.(Gly416Ser)	A 21-year-old son
					D030	*TNNT2*	NM_001276345.2:c.506G>A	NP_001263274.1:p.(Arg169Gln)	A 2-year-old son
Overall	62	36	66	5 (7.58%)					

## Discussion

### Current status of the inherited cardiovascular condition service in developing countries

ICC clinics aim to provide diagnoses of certain inherited cardiovascular conditions, management, genetic counseling, genetic testing, and screening of asymptomatic family members. Such clinics require a multidisciplinary team that specializes in different fields. The most common referrals to such clinics are cardiomyopathy (HCM, DCM, arrhythmogenic cardiomyopathy, and left ventricular noncompaction), arrhythmia (Brugada syndrome and long QT syndrome), aortopathy (Marfan syndrome and nonsyndromic thoracic aneurysm), and pulmonary hypertension cases. Several ICC clinics around the world detect new cases in the family, with the aim of offering early intervention to prevent morbidity and mortality. Establishing an ICC clinic in a developing country is a great challenge because genetic testing is costly and requires government subsidies. For example in Thailand, the gross domestic product (GDP) per capita was 6,450 USD in 2020 (data obtained from the World Bank Organization), but next-generation sequencing performed domestically was estimated to be less than 1,160 USD. In addition, the number of genetic professionals, such as cardiologists specializing in ICC, clinical geneticists, clinical laboratory geneticists, bioinformaticians, and genetic counselors, is limited. The ratio of clinical geneticists per 100,000 people in Thailand is 0.04. These socioeconomic parameters make it difficult to establish ICC clinics in the country [5]. Currently, our single-center ICC clinic is at the toddler stage, and the sample size provided in this registry may not represent the situation of the whole nation. In addition, our relatively small sample size had its effects on the statistical analysis. If we could increase the number of ICC professionals around the country, it would be fantastic to initiate multi-center collaboration and improve accurate national statistics.

### Molecular diagnosis of hypertrophic cardiomyopathy

To date, pathogenic variants causing HCM have been characterized in one of the genes encoding sarcomere proteins, with *MYBPC3* and *MYH7* being the most prevalent [42,43]. Variants in the sarcomere gene have been identified in 50–60% of patients with family history and in 20–40% of patients with sporadic HCM [44,45]. Our results showed that all variants (P/LP/VUS) were identified in 9 of 16 patients (56.25%) with family history and in 6 of 15 patients (40%) with no significant family history, thereby corroborating the results of previous studies.

In terms of the distribution amongst age groups, classic HCM is commonly seen in adolescents. Nevertheless, the average and median age of onset of patients in our registry were up to 50 years. In fact, our joint ICC clinic was established under the adult cardiology service, which accepts a referral of patients above 15 years of age. Therefore, younger patients under pediatric care were not included in this study. Furthermore, our cohort displayed a high proportion of variants detected in *MYBPC3*, which has been associated with a later onset age [46]. Meanwhile, the lowest onset age of patients in our ICC registry who carried an *MYH7* pathogenic variant was 16 years (H016) (S1 Table). *MYH7* is a well-known gene as a cause of HCM in younger patients, resulting in significant LVH and the development of symptoms by the second decade of life [47]. Owing to the lack of younger patients in our department, the proportion of *MYH7*-associated HCM in our cohort seemed to be lower than the general incidence.

However, approximately 16% of patients carried VUS owing to insufficient bioinformatic data to support pathogenic characteristics, that is, variants in *ACTN2* (H017*)*, *MYBPC3* (H018), *TPM1* (H003), and *VCL* (H011) (S1 Table). These VUS were described as missense and displayed the potential of pathogenic criteria owing to various bioinformatic information, such as the absence of minor allele frequency in controls, *in silico* analysis suggestive of protein damage, and location in highly conserved regions. However, the total interpretation scores did not meet the P/LP criteria and required further family studies. Familial co-segregation analysis data would be highly informative if an apparent autosomal dominant inheritance or suggestive *de novo* occurrence was elucidated. A recent study demonstrated that novel variant detection was estimated at 35–40%, and 56% were considered “private” variants specific to each family [44]. Although data retrieved from familial co-segregation analysis would be helpful to convert these VUS into P/LP, family tracking was difficult for several reasons, such as none of the living relatives, relatives living in a different city, and asymptomatic relatives showing no interest in genetic testing.

Most P/LP variants in our HCM study were predominantly missense variants, resulting in nonsynonymous amino acid substitutions. Therefore, the variant peptides encoded by those heterozygous genomic variants might negatively interfere with the co-expressed wild-type protein. This phenomenon suggests a dominant-negative effect [48]. However, not all missense variants contribute to this phenomenon. The dominant-negative effect usually occurs if the variant product of a particular gene can only interact with the same elements as a wild-type product and adversely affect the normal protein function [49]. In the meantime, nearly half of the variants in *MYBPC3* in our study were caused by frameshifts and splice-site variants, suggesting a loss-of-function and haploinsufficiency mechanism [50].

Several clinical predictors have been proposed to be associated with the presence of genetic variants in HCM patients. We performed statistical analysis for age at onset, left ventricular obstruction, arrhythmias, syncope, and family history. We demonstrated that only a median age of less than 52 years at onset and left ventricular obstruction were statistically significant. Previous publications have described that the variables related to a higher probability of a positive genetic test included family history, young age, left ventricular thickness, heart failure, and ventricular arrhythmia [51–53].

### Molecular diagnosis of dilated cardiomyopathy

The etiology of DCM is diverse and can be categorized as acquired, syndromic, or non-syndromic. Our ICC registry included only patients diagnosed with idiopathic DCM without other systemic involvement; therefore, the most well-known acquired causes were excluded, such as ischemic process, a significant history of acute viral myocarditis, particular drug use, heavy alcohol consumption, chronic kidney disease stage 4–5, hyperthyroidism, uncontrolled hypertension, and other suspicious conditions. Patients with syndromic DCM, such as cardiomyopathy found in neuromuscular disorders, neurodevelopmental disorders, and inherited metabolic diseases, were also excluded. The overall variant detection rate, including P/LP/VUS, was approximately 25.81% (P/LP16.13%; VUS 9.68%). Focusing on the probability of P/LP variant detection and the family history correlation, the culprit variants were identified in 2 of 9 patients (22.22%) with family history and 3 of 22 patients (13.64%) with no family history (S2 Table). These results are consistent with those of previous literature describing the identification of a culprit variant in approximately 20–40% of patients with familial DCM and approximately 13–25% of patients with sporadic DCM [54,55].

To date, the most prevalent genes contributing to the DCM phenotype include *TTN* (15–20%), *LMNA* (6%), *MYH7* (4%), *FLNC* (2–4%), *BAG3* (3%), and *TNNT2* (3%) [56]. The other reported genes are rare. The other reported genes are rare. Our ICC registry demonstrated *TTN*, *MYH7*, *MYBPC3*, and *TNNT2* as P/LP, but VUS was also detected in *MYH7*, *SCN5A*, and *CSRP3*. *TTN* encodes a giant protein called Titin, which is responsible for the passive elasticity of cardiac muscle, and this gene is known to be highly variated [57]. Many *TTN* variants were obtained following NGS data generation and classified as benign.

Similar to the approach in the HCM group, familial co-segregation analysis would be helpful to confirm the pathogenicity of VUS. Variants causing DCM are also suggestive of either dominant-negative or haploinsufficient mechanisms.

In our cohort, none of the clinical predictors was related to the presence of genomic findings, that is, age at onset, LVEF, arrhythmias, ICD implantation, and family history. However, to facilitate screening, genetic testing is recommended for all patients with familial DCM. In contrast, guideline recommendations for testing in patients with sporadic DCM differ, but specific clinical features might increase the testing yield [54].

### Cardiac and genetic surveillance in first-degree relatives

The results of our study suggest that the offer for cardiac screening in first-degree relatives of patients diagnosed with HCM or DCM is still beneficial, even though the genomic variant is either present or absent. We established a genetic counseling system for relatives of all newly diagnosed cases by issuing referral letters to all first-degree relatives. The letter mentions why they needed cardiovascular surveillance and genetic testing. Most developing countries face similar difficulties because the national genetic counseling system is in the toddler stage [8]. This work is currently consultant-led and varies based on the individual’s practice. However, 60% of the relatives in our cohort who received our letter robustly followed the recommendations within the first year of contact.

The detection rate of echocardiogram surveillance and genetic testing among asymptomatic relatives was attractive. We detected 8.82% and 6.25% new presymptomatic cases of HCM and DCM, respectively. Thus, our genetic service detected new patients, approximately one in 13 asymptomatic family members (Table 2). Previous studies also showed that a new diagnosis for HCM in children’s first-degree relatives was approximately 8–10% [58]. Conclusively, family screening remains essential, and relatives would benefit from early detection and intervention to prevent subsequent adverse outcomes. Although the ICC system in developing countries is not solid, genetic testing and family screening are recommended to apply clinical practice guidelines worldwide, based on each national healthcare context [59,60].

## Conclusions

This research highlighted the lessons and learning curve of genomic findings of non-syndromic HCM and idiopathic DCM in ICC clinics in a developing country. We provided genomic data sharing from the Southeast Asian population, which would be useful for the global database. The national ICC registry in our country remains at a beginner stage. Our aim is to introduce these data to the government to consider the reimbursement of genetic testing for cardiomyopathy in patients and their relatives. In addition, we need national-level support to train more staff members keen to practice ICC and increase awareness in other clinicians about the genetic referral of these conditions.

## Supporting information

S1 Table Clinical information of patients affected by hypertrophic cardiomyopathy in this study. (PDF)

S2 Table Clinical information of patients affected by dilated cardiomyopathy in this study. (PDF)
