# Supplementary material for: Genomic findings of hypertrophic and dilated cardiomyopathy characterized in a Thai clinical genetics service
Source: PLoS One. 2022 Sep 27;17(9):e0267770. doi: 10.1371/journal.pone.0267770 (PMC9514623; doi:10.1371/journal.pone.0267770)
Supplement: S1 Table — (PDF) [file pone.0267770.s001.pdf]

S1 Table. Clinical information of patients affected by hypertrophic cardiomyopathy in this study.

ID	Sex	Age of Onset	LV Obstruction	Unexplained Syncope	Arrhythmia[a]	ICD Implantation	Family History[b]	Gene	HGVS Coding DNA	HGVS Coding Protein	Classification
H001	M	70	Y	N	Y	N	Y	ND	[redacted]	[redacted]	[redacted]
H002	M	49	N	N	Y	N	N	*MYBPC3*	NM_000256.3:c.1522C>T	NP_000247.2:p.(Gln508Ter)	Pathogenic
H003	M	25	Y	N	Y	N	N	*TPM1*	NM_000366.6:c.343G>A	NP_000357.3 :p.(Glu115Lys)	VUS
H004	F	43	N	N	Y	N	N	ND	[redacted]	[redacted]	[redacted]
H005	F	33	Y	N	N	N	Y	*MYBPC3*	NM_000256.3:c.3624_3624delC	NP_000247.2:p.(Lys1209fs)	Pathogenic
H006	F	67	N	N	Y	N	Y	ND	[redacted]	[redacted]	[redacted]
H007	M	66	N	N	N	N	Y	ND	[redacted]	[redacted]	[redacted]
H008	M	59	N	N	N	N	N	ND	[redacted]	[redacted]	[redacted]
H009	M	58	N	N	N	N	N	ND	[redacted]	[redacted]	[redacted]
H010	M	68	Y	N	Y	N	Y	*MYBPC3*	NM_000256.3:c.2864_2865delCT	NP_000247.2:p.(Pro955fs)	Pathogenic
H011	M	42	Y	N	N	N	Y	*VCL*	NM_003373.4:c.833A>G	NP_003364.1 :p.(Asn278Ser)	VUS
H012	M	47	Y	Y	Y	Y	N	*MYBPC3*	NM_000256.3:c.2864_2865delCT	NP_000247.2:p.(Pro955fs)	Pathogenic
H013	M	61	Y	N	N	N	N	ND	[redacted]	[redacted]	[redacted]
H014	M	37	N	N	N	N	Y	*MYBPC3*	NM_000256.3:c.2300A>G	NP_000247.2:p.(Lys767Arg)	Likely pathogenic
H015	F	71	Y	N	Y	N	N	*MYBPC3*	NM_000256.3:c.1058delA	NP_000247.2:p.(Lys353Argfs*3)	Pathogenic
H016	M	16	Y	N	N	N	Y	*MYH7*	NM_000257.4:c.2146G>A	NP_000248.2:p.(Gly716Arg)	Pathogenic
H017	F	52	Y	N	N	N	N	*ACTN2*	NM_001103.4:c.1586A>G	NP_001094.1:p.(Asn529Ser)	VUS
H018	M	41	Y	N	Y	Y	Y	*MYBPC3*	NM_000256.3:c.3190+5G>A	[redacted]	Pathogenic
H019	M	64	N	N	N	N	Y	ND	[redacted]	[redacted]	[redacted]
H020	M	52	N	N	N	N	Y	ND	[redacted]	[redacted]	[redacted]
H021	M	27	N	N	N	N	Y	ND	[redacted]	[redacted]	[redacted]
H022	M	57	Y	N	N	N	N	ND	[redacted]	[redacted]	[redacted]
H023	M	42	Y	Y	Y	Y	N	*MYL2*	NM_000432.4:c.173G>A	NP_000423.2:p.(Arg58Gln)	Pathogenic
H024	M	82	N	N	N	N	N	ND	[redacted]	[redacted]	[redacted]

H025	M	66	N	N	Y	N	N	ND			
H026	M	61	N	N	N	N	Y	*MYBPC3*	NM_000256.3:c.1720C>T	NP_000247.2:p.(Arg574Trp)	VUS
H027	M	46	Y	N	N	N	Y	ND			
H028	F	45	Y	Y	Y	Y	Y	*TNNI3*	NM_000363.5:c.370G>C	NP_000354.4:p.(Glu124Gln)	Pathogenic
H029	F	50	Y	N	Y	Y	Y	*MYBPC3*	NM_000256.3:c.1144C>G	NP_000247.2:p.(Arg382Gly)	VUS
H030	F	70	Y	N	Y	Y	N	ND			
H031	F	77	Y	N	Y	Y	N	ND			

[a]The types of arrhythmia identified in the population of this study included atrial fibrillation, supraventricular tachycardia, ventricular tachycardia, and ventricular fibrillation.

[b]The family history was considered positive when one of the first-or second-degree relatives was diagnosed with hypertrophic cardiomyopathy or sudden unexplained cardiac death.

Abbreviations: M, male; F, female; LV, left ventricle; ICD, implantable cardioverter-defibrillator; Y, yes; N, no; ND, not detected; VUS, variant of uncertain significance.
