# Supplementary material for: Genomic findings of hypertrophic and dilated cardiomyopathy characterized in a Thai clinical genetics service
Source: PLoS One. 2022 Sep 27;17(9):e0267770. doi: 10.1371/journal.pone.0267770 (PMC9514623; doi:10.1371/journal.pone.0267770)
Supplement: S2 Table — (PDF) [file pone.0267770.s002.pdf]

S2 Table. Clinical information of patients affected by dilated cardiomyopathy in this study.

ID	Sex	Age of Onset	LVEF (%)	Arrhythmia[a]	ICD Implantation	Family History[b]	Gene	HGVS Coding DNA	HGVS Protein	Classification
D001	M	25	45	Y	N	Y	ND	[redacted]		
D002	M	43	40	N	N	N	ND			
D003	F	46	44	N	N	N	ND			
D004	M	53	25	Y	Y	N	ND			
D005	M	54	21	Y	Y	N	ND			
D006	F	40	25	N	N	N	ND			
D007	F	57	20	Y	N	N	ND			
D008	M	30	20	Y	Y	N	ND	[redacted]		
D009	M	34	18	Y	N	N	ND			
D010	M	71	40	Y	N	Y	ND			
D011	M	53	24	Y	Y	N	ND			
D012	M	44	20	Y	Y	N	*TTN*	NM_001267550.2:c.85493G>A	NP_001254479.2 :p.(Trp28498Ter)	Pathogenic
D013	M	56	24	N	N	Y	ND	[redacted]		
D014	M	67	20	Y	N	N	*MYH7*	NM_000257.4:c.3157C>T	NP_000248.2:p.(Arg1053Trp)	Pathogenic
D015	M	63	45	Y	N	N	ND	[redacted]		
D016	F	26	45	N	N	N	ND			
D017	M	54	25	Y	Y	N	ND			
D018	M	30	42	N	N	N	ND			
D019	M	47	35	Y	N	Y	*SCN5A*	NM_000335.5:c.677C>T	NP_000326.2 :p.(Ala226Val)	VUS
D020	M	42	37	N	N	Y	ND	[redacted]		
D021	M	19	20	Y	Y	N	ND			
D022	F	46	41	N	N	Y	*MYBPC3*	NM_000256.3:c.1246G>A	NP_000247.2:p.(Gly416Ser)	Likely pathogenic
D023	F	54	35	Y	N	Y	*TTN*	NM_001256850.1:c.71731C>T	NP_001243779.1: p.(Arg23911Ter)	Pathogenic
D024	F	19	29	N	N	N	ND	[redacted]		

D025	F	16	40	N	N	N	ND	[redacted]			
D026	M	37	38	N	N	N	ND	[redacted]			
D027	F	46	40	Y	N	Y	ND	[redacted]			
D028	M	46	45	Y	N	N	ND	[redacted]			
D029	M	25	20	Y	Y	N	*MYH7*	NM_000257.4:c.4298A>G	NP_000248.2:p.(Glu1433Gly)	VUS
D030	M	39	23	Y	Y	N	*TNNT2*	NM_001276345.2:c.506G>A	NP_001263274.1:p.(Arg169Gln)	Likely pathogenic
D031	F	44	25	Y	Y	Y	*CSRP3*	NM_003476.5:c.571G>A	NP_003467.1:p.(Glu191Lys)	VUS

[a]The types of arrhythmia identified in the population of this study included atrial fibrillation, supraventricular tachycardia, ventricular tachycardia, and ventricular fibrillation.

[b]The family history was considered positive when one of the first- or second-degree relatives was diagnosed with dilated cardiomyopathy, sudden unexplained cardiac death, or an unknown cause of congestive heart failure.

Abbreviations: M, male; F, female; Y, yes; N, no; LVEF, left ventricular ejection fraction; ND, not detected; VUS, variant of uncertain significance.
